# The Art of Planning a Left Atrial Appendage Closure: Will Computational Modeling Simplify the Intervention?∗

**DOI:** 10.1016/j.jacadv.2022.100138

**Published:** 2022-11-30

**Authors:** Kasper Korsholm, Jens Erik Nielsen-Kudsk

**Affiliations:** Department of Cardiology, Aarhus University Hospital, Aarhus N, Denmark

**Keywords:** cardiac computed tomography, computational modelling, left atrial appendage occlusion, Watchman

**Figure:**
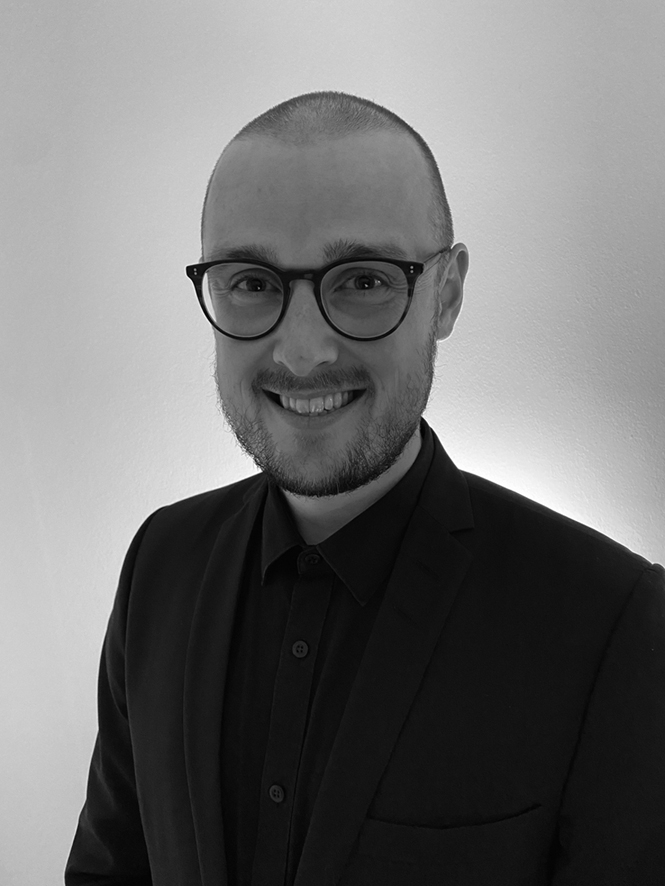

Transcatheter left atrial appendage occlusion (LAAO) in atrial fibrillation has undergone major advancements since the pivotal PROTECT-AF (Watchman Left atrial appendage system for embolic PROTECTion in patients with Atrial Fibrillation) and PREVAIL (Prospective Randomized evaluation of the Watchman LAA closure device in patients with atrial fibrillation versus long term warfarin therapy) trials demonstrated noninferiority compared to warfarin.^1^ Despite the growing evidence supporting its efficacy and safety, technical limitations remain. It is increasingly recognized that the technical result of a LAAO procedure will affect the long-term outcome. Peridevice leak appears to increase the risk of thromboembolic complications. The mechanism for leaks is often malaligned devices, with low device compression, larger left atrial appendages (LAAs), or complex anatomy being a predisposing factor. Implant depth (>10 mm from the left upper pulmonary vein ridge [LUPV]) has shown association to device-related thrombosis. This has led to a focus on improving the LAAO technique by addressing the risk of residual peridevice leak and device-related thrombosis to further improve clinical outcomes.

The field of interventional cardiac imaging has also made a major leap forward in recent years. The enormous variation in the size, shape, and orientation of the LAA makes careful preprocedural planning essential to anticipate intraprocedural challenges and attempt to predict how the device will interact with the LAA anatomy. It has for many years been confined to 2-dimensional transesophageal echocardiography (TEE), which was used in the pivotal trials but was later shown to be inferior to 3-dimensional (3D) sizing.^2^^,^^3^ Currently both 3D-TEE and cardiac computed tomography (CT) are widely used, but the latter has the obvious advantages of being noninvasive and highly patient-friendly, while providing isotropic data with high spatial resolution allowing for multiplanar and 3D reconstruction with further possibilities to do advanced preprocedural planning with 3D printing or computational modeling.^3^

In this issue of *JACC: Advances*, Ranard et al^4^ provide a study focused on preprocedural planning with artificial intelligence-enhanced computational modeling using the FEops HEARTguide. Based on the combination of digital twins with artificial intelligence-enabled anatomical analyses, FEops provides LAAO implant simulations with the goal to let the operator visualize different device sizes positioned at different implantation depths. A color map of wall apposition allows a visual assessment of potential peridevice leak. Ranard et al^4^ present a retrospective analysis on the accuracy of the FEops HEARTguide model to predict device size, location, and compression in 22 patients undergoing LAAO with the WATCHMAN FLX device. Patients had preprocedural and postprocedural cardiac CT available, and LAAO procedures were guided by intraprocedural 3D-TEE. LAAO operators were unaware of the FEops model results at the time of the LAAO procedure, and no complications or peridevice leak were noted at follow-up.

To evaluate the accuracy of the FEops model, the authors performed a blinded and unblinded simulation of the device implant result using the FEops HEARTguide platform. In the blinded analysis, the preprocedural cardiac CT was uploaded to FEops without information on the actual implanted device size or implantation result. In the secondary unblinded analysis, FEops was knowledgeable about device size and was provided with the postprocedural CT to see if the model was able to predict the actual implant result. Twenty-five preprocedural CT scans were uploaded, and 3 of these had insufficient quality for computational modeling.

The FEops model predicted the actual implanted device size in 16 of 22 (72.7%) patients, and 13 (59% of investigated LAAO cases) had a simulated device position that was considered comparable to that in the actual postprocedural CT. The size was comparable but the position was not in 3 (18.7%) patients. The baseline LAA dimensions and device compression measures were comparable between the FEops model and intraprocedural 3D-TEE, with a strong correlation (Pearson correlation coefficient >0.90). The FEops model did not include the actual implanted device size in 6 (27.3%) patients, and these were excluded from the device compression and position comparison. All 6 patients received a larger device size than predicted by FEops, and the position was more proximal than predicted by the FEops model. The unblinded analysis excluded 1 postprocedural CT scan due to insufficient image quality, while the FEops model could be modified to predict the actual device size and provided comparable device compression measures in the remaining patients.

When interpreting the study results, it is key to remember that more than 1 device may be appropriate and able to achieve a successful LAAO result. Optimal compression of the WATCHMAN FLX device is between 10% and 30%; hence, sizing charts includes a certain degree of overlap of devices suitable for various LAA dimensions. Device position evaluation should be relative to the anatomical landmarks of the left circumflex coronary artery and the LUPV, defining the orifice of the LAA. Less than one-third of the device length may protrude proximal of a line connecting the 2 anatomical landmarks, and the device should be <10 mm distal to the LUPV ridge as it may increase the risk of device-related thrombosis during follow-up. This leaves a certain range of acceptable device sizes and positions considered as optimal for certain LAA anatomies. Disagreement was present on device size in 6 patients and on device position in 3 patients. Overall, actual implant result and the FEops model disagreed in 41% of cases (9/22). Does this make the model accurate at predicting actual device implant characteristics? Not particularly based on an agreement limited to 59% of cases. But does this make the FEops model poor at predicting the optimal LAAO implant result? Not necessarily. The authors allude to this and state that after a second review, they believe the FEops predictions could have led to successful LAAO in all cases.

This highlights a key limitation to the study; the lack of a gold standard to compare the results against. Core-Lab analysis or adjudication of imaging and simulations were not performed. The Pearson correlation coefficients uniformly indicated a very good correlation between LAA dimensions and postimplant device dimensions assessed by the FEops model and intraprocedural TEE. It should be acknowledged that correlation not necessarily means agreement, but measures were not statistically different. The comparison is, however, limited by exclusion of cases with disagreement on device size, which intuitively leads to better correlation and agreement between methods. Finally, the study design is associated with the inherent limitations of a small, retrospective study. Regardless, the study leaves the reader with the important question whether knowledge of the FEops simulation results would have changed the choice of device or led to repositioning if performed prospectively. No complications or peridevice leak was noted in the 22 patients, but whether the FEops computational modeling will improve LAA sealing or clinical outcomes remain undetermined and should be addressed in future trials.

Preprocedural planning of LAAO may be perceived as an artform requiring the operator to imagine the potential implant result—but like art, different interpretations may be concluded by different observers, and it does not always reflect reality. Many factors will affect the final procedural outcome: transseptal puncture site, delivery sheath shape, LAA orientation, degree of LAA trabeculation, experience of the LAAO operator/team, and so on. The use of computational modeling is promising, and in practice, the models provide the operator with various device size options at different implantation depths (proximal and distal). The visual assessment allows the operator to come closer for actual visualization of the technical outcomes achievable by different devices at different positions, which may aid decision-making. Computational modeling may prove particularly useful in cases where standard sizing charts indicate 2 devices may be equally good. The highly heterogenous LAA anatomy makes LAAO particularly complex and demands careful and advanced preprocedural planning to achieve optimal results. The present study motivates the need for a future randomized trial to evaluate if there is an added value of preprocedural planning with the FEops HEARTguide over a standard cardiac CT with device sizing based on multiplanar views and 3D rendering of the LAA anatomy. A prospective trial (PREDICT-LAA [The value of FEops HEARTguide Patient-specific computational simulation in the planning of percutaneous left atrial appendage closure with the Amplatzer Amulet device]; NCT04180605) is currently ongoing and enrolling patients planned to undergo Amplatzer Amulet (Abbott) LAAO, with the aim to evaluate the impact of computational modeling on the primary outcome of incomplete LAA closure and device-related thrombosis assessed by 3-month postprocedural cardiac CT.^5^

## Funding support and author disclosures

Dr Korsholm has received speaker’s honorarium from Abbott and Boston Scientific; and has received unrestricted institutional education grants from both 10.13039/100000046Abbott and 10.13039/100008497Boston Scientific. Dr Nielsen-Kudsk is proctor for Abbott and Boston Scientific; and has received unrestricted institutional research grants from 10.13039/100000046Abbott and 10.13039/100008497Boston Scientific. Both authors are investigators in the PREDICT-LAA trial.
